# Topological classification of tumour-immune interactions and dynamics

**DOI:** 10.1007/s00285-025-02253-6

**Published:** 2025-08-05

**Authors:** Jingjie Yang, Heidi Fang, Jagdeep Dhesi, Iris H. R. Yoon, Joshua A. Bull, Helen M. Byrne, Heather A. Harrington, Gillian Grindstaff

**Affiliations:** 1 Mathematical Institute, University of Oxford, Oxford, OX2 6GG UK; 2 Wesleyan University, Middletown, CT 06459 USA; 3 Ludwig Institute for Cancer Research, University of Oxford, Oxford, OX3 7DQ UK; 4 Max Planck Institute Center for Systems Biology, Dresden, Germany

## Abstract

The complex and dynamic crosstalk between tumour and immune cells results in tumours that can exhibit distinct qualitative behaviours—elimination, equilibrium, and escape—and intricate spatial patterns, yet share similar cell configurations in the early stages. We offer a topological approach to analyse time series of spatial data of cell locations (including tumour cells and macrophages) in order to predict malignant behaviour. We propose four topological vectorisations specialised to such cell data: persistence images of Vietoris-Rips and radial filtrations at static time points, and persistence images for zigzag filtrations and persistence vineyards varying in time. To demonstrate the approach, synthetic data are generated from an agent-based model with varying parameters. We compare the performance of topological summaries in predicting—with logistic regression at various time steps—whether tumour niches surrounding blood vessels are present at the end of the simulation, as a proxy for metastasis (i.e., tumour escape). We find that both static and time-dependent methods accurately identify perivascular niche formation, significantly earlier than simpler markers such as the number of tumour cells and the macrophage phenotype ratio. We find additionally that dimension 0 persistence applied to macrophage data, representing multi-scale clusters of the spatial arrangement of macrophages, performs best at this classification task at early time steps, prior to full tumour development, and performs even better when time-dependent data are included; in contrast, topological measures capturing the shape of the tumour, such as tortuosity and punctures in the cell arrangement, perform best at intermediate and later stages. We analyse the logistic regression coefficients for each method to identify detailed shape differences between the classes.

## Introduction

The ecosystem of cells that surround a tumour - the tumour micro-environment - is extremely complex. The interplay of tumour cells with immune cells and the surrounding blood vessels and tissue plays a crucial role in the evolution of the tumour micro-environment (Elmusrati et al. 2021). For example, interactions with the tumour can lead the immune cells to either support or inhibit tumour cell proliferation and migration (Junttila and De Sauvage 2013). An overarching goal in mathematical oncology is the study and prediction of tumour behaviour from experimental observations, which may improve understanding of the complex system and inform treatment strategies. With advances in technologies and more sophisticated experimental models, spatio-temporal data of species in the tumour microenvironment are more readily available (Lewis et al. 2021; Tian et al. 2023). In addition to experimental data, we can generate synthetic data by simulating agent-based models that encode species interactions in the tumour microenvironment. In contrast to biological imaging data which is typically only collected at a single time point, the agent-based models can provide data about species locations at multiple time points and under multiple different conditions. We propose the mathematical machinery of *persistent homology* to quantify the spatio-temporal evolution of tumour and immune species. We compare the spatio-temporal analysis using topological descriptors with simpler descriptors, such as tumour size, macrophage phenotype ratio and minimum distance between tumour and blood vessel. Our aim is to analyse, classify and predict different cellular behaviours with direct consequence to tumour outcome.

Researchers have long known that innate immune cells such as macrophages can exhibit both pro- and anti-tumour behaviours depending on their phenotype (Gonzalez et al. 2018). The relationships between the spatial and phenotypic distributions of macrophages and patterns of tumour growth are believed to hold great significance for prognosis (Cortese et al. 2020) and responses to cancer immunotherapy (Long and Beatty 2013). Many models of macrophage-tumour interactions have been developed (eg, Owen and Sherratt 1999; Kelly et al. 1900; Owen et al. 2004; Li et al. 2019; Den Breems and Eftimie 2016; El-Kenawi et al. 2019), including models of the spatial interactions between macrophages and tumour cells required for the development of perivascular niches (such as the CSF-1/EGF paracrine loop driving cross-talk between pro-tumour macrophages and tumour cells (Knútsdóttir et al. 2014, 2016; Elitas and Zeinali 2016)) and models which resolve macrophage phenotype as a continuum (e.g. Eftimie 2020; El-Kenawi et al. 2019). Here we will restrict analysis to synthetic data generated previously from a two-dimensional, off-lattice *agent-based model* (ABM) by Bull and Byrne (2023), in which a wide range of tumour-macrophage interactions can occur as the macrophage phenotype varies. The ABM distinguishes four cell types—macrophages, stromal, tumour, and necrotic cells. Each cell is represented by an agent whose behaviour is determined by a set of rules: each cell is subject to mechanical and chemotactic forces exerted by neighbouring cells, and the net force determines how its spatial position changes over time.

**Fig. 1:**
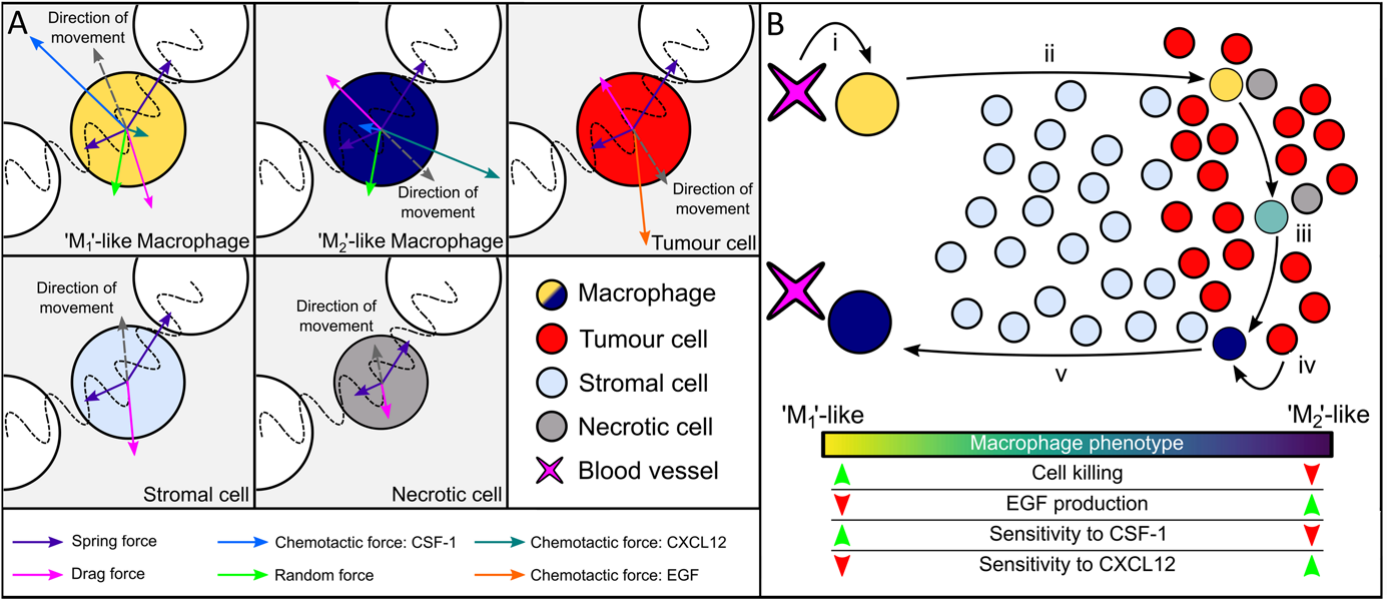
Schematic of the ABM. A: Each cell acts as an individual agent, with activity depending on the physical effect of surrounding cells and local levels of diffusible species CSF-1, EGF, or CXCL12. Macrophages have two phenotypes with different behaviours: M_1_ (anti-tumour) and M_2_ (pro-tumour). B: Summary of the tumour-immune dynamics i) M_1_ macrophages extravasate from blood vessels in response to CSF-1 produced by tumour cells. ii) M_1_ macrophages migrate into the tumour mass in response to CSF-1, where they may kill tumour cells on contact (these then become necrotic cells). iii) Exposure to TGF-$\beta$ causes macrophages to adopt an M_2_ phenotype. iv) M_2_ macrophages produce EGF, which acts as a chemoattractant for tumour cells. v) M_2_ macrophages migrate towards blood vessels, in response to CXCL12 gradients, with tumour cells following the EGF gradient behind. Reproduced from Bull and Byrne (2023), Fig 2

Macrophage phenotype is modelled as a continuous subcellular variable $p_i \in [0, 1]$: $p_i = 0$ represents an anti-tumour ‘M_1_’ phenotype, and $p_i = 1$ represents a pro-tumour ‘M_2_’ phenotype, whilst a macrophage with an intermediate phenotype exhibits intermediate behaviour. As tumour cells release the diffusible chemoattractant *colony stimulating factor-1* (CSF-1), M_1_-like macrophages with phenotype $p_i = 0$ extravasate from blood vessels and move towards tumour cells to kill them. Under sufficient exposure to *transforming growth factor beta* (TGF-$\beta$) in the tumour microenvironment, the macrophages adopt an M_2_-like phenotype and migrate towards nearby blood vessels, guiding the migration of their neighbouring tumour cells. If sufficient numbers of tumour cells migrate with the M_2_ macrophages all the way to a blood vessel, a *perivascular niche* of tumour cells may be formed. This is summarised in Fig. 1, where we present a schematic of the model.

Once tumour cells accumulate and proliferate around blood vessels to form a perivascular niche, the likelihood of tumour intravasation and subsequent metastasis naturally increases. Indeed, such microenvironments are regarded as “propulsion centres of the tumour” (Laura et al. 2017). There are various proposed methods for quantifying and studying shape of such cellular behaviours. For example, tumour cell counts and the minimum tumour-vessel distance provide a reasonable indicator of the presence of perivascular niches at the end of the simulation (see Sec. 4.4), but subtle shape changes may allow for prediction at earlier stages before the tumour approaches the vessel or gets eliminated. Other spatial statistics such as the pair-correlation function have been applied to study cellular heterogeneity and organisation (Bull et al. 2020; Bull and Byrne 2023), but analysis of cell locations.

*Topological data analysis* (TDA) is a growing field of computational mathematics focused on understanding the fundamental shape and structural features inherent in complex data sets. In recent years, there has been a wide range of research applying TDA to biomedical data to quantify differences in shape and behaviour, as described in surveys such as Amézquita et al. (2020); Rabadan and Blumberg (2019), and even, as we do in this work, to study an agent-based model of immune cell infiltration into a tumour-spheroid (Vipond 2020). At its core, TDA captures multi-scale structural characteristics of data - clusters, loops, and other higher-dimensional features - providing unique insights beyond traditional methods (Carlsson 2009).

To accomplish this, TDA employs mathematical structures called simplicial complexes (see e.g. Fig. 5), which can be viewed as generalizations of networks capable of encoding multi-dimensional relationships among data points. The key analytical technique within TDA, *persistent homology* (PH), systematically tracks how the topology of a 1-parameter family of simplicial complexes (also called a *filtration*) evolves as the parameter, for example scale, density, time, or phenotypic threshold, varies (see e.g. Fig. 4). Persistent homology thus transforms the data into algebraic summaries that highlight structural changes with respect to a chosen parameter.

The strength of persistent homology lies in its deep theoretical foundations in algebraic topology and stability results that facilitate a wide range of statistics (Cohen-Steiner et al. 2006; Kim et al. 2019; Chazal et al. 2009, 2021; Fasy et al. 2014; Tukey and Donoho 2022), along with its radical adaptability for different data types and shapes (Stolz-Pretzer 2019). Here, a series of choices propagate through the persistent homology pipeline: data $\rightarrow$ filtered simplicial complex $\rightarrow$ persistent homology $\rightarrow$ analysis and interpretation (see Figs. 4, 5, 6 and 7). In practice, constructing the appropriate filtration is particularly important since different filtrations capture different geometric and biological features of the data. Furthermore, since the resulting persistence diagrams form complex mathematical spaces lacking conventional averages or inner products, effective statistical analysis typically involves converting these diagrams into vector representations (Ali et al. 2023). We use the persistence image vectorization for all analyses (Adams et al. 2017), as it has been observed to work well in machine learning tasks.

As the data have multiple cell types, with both spatial and temporal features, we make several different choices of emphasis in our filtrations and compare the results. The widely used *(Vietoris-)Rips filtration* (VR) is formed from multi-scale distances between data points. In the past few years, studies such as Lawson et al. (2019); Bhaskar et al. (2021); Bussola et al. (2021); Vandaele et al. (2023) have successfully applied VR features for machine-learning classification of real cell configurations, including histology slides of tumours in situ. Furthermore, VR features computed from multiple cell types have been used to classify spatial patterns arising from simulations of an agent-based model (Bhaskar et al. 2022).

Besides distance-based filtrations like Vietoris-Rips, more bespoke filtrations have been proposed in other applications. For example, the *radial filtration* we use has been previously applied to neuronal trees and vascular networks (Kanari et al. 2018; Stolz et al. 2022; Nardini et al. 2023). In this filtration, distance from a central basepoint replaces pairwise distances of cell locations; this encodes the boundary tortuosity, components, and holes according to their radius and reach (Bubenik et al. 2020).

Incorporating the dynamics of spatial cell data requires more advanced (and computationally expensive) techniques. Topaz et al. (2015) analyse agent-based models of biological aggregations using a VR filtration for each snapshot of a dynamical system, and track the variation of real-valued summary statistics as time evolves to create topological time series data. The *persistence vineyard* is another variant; it tracks the temporal variation of a VR complex directly in the persistence diagrams (Cohen-Steiner et al. 2006). Persistence vineyards have been recently applied to find regional anomalies of COVID-19 cases and functional connectivity of the brain (Hickok et al. 2021; Yoo et al. 2016). A related summary, the Crocker matrix, has been applied to the D’Orsgona model for spatial cell interactions (Nguyen et al. 2024). These methods allow for an approximation of the natural continuity between topological disruption events.

Computing topological persistence over time using data continuity directly requires a different approach due to non-monotonicity of the corresponding complex. *Zigzag persistence* (Carlsson and De Silva 2010) was developed theoretically alongside scale-parameter methods, and it tracks the evolution of individual features directly rather than the diagram as a whole by substituting time scale for distance. Zigzag PH has been applied to analyse complex systems (Corcoran and Jones 2016), find Hopf bifurcations (Tymochko et al. 2020), compare coral reef models (McDonald et al. 2022), and extract higher-dimensional structure from 2D images (Mata et al. 2015).

In this work, we implement two variations each of static and dynamic methods and apply them to spatial data from Bull and Byrne’s agent-based model of tumour-immune interaction. From the spatial locations of tumour cells, macrophages, and blood vessels, we demonstrate four approaches in total: the Vietoris-Rips and radial filtrations quantifying the shape of the cell point cloud at individual timesteps, as well as the persistence vineyards and zigzag persistence tracking the evolution of cell clusters through the simulation. We show that these techniques give complementary, interpretable spatio-temporal information, and we compare their predictive power by training a logistic regression classifier on each to detect patterns which predict the formation of perivascular niches in simulated data. We show significant improvements over benchmark statistics, including higher accuracy and earlier classification than macrophage phenotype ratio, tumour cell count, and tumour proximity to blood vessels. Additionally, we interpret the logistic regression coefficients to identify qualitative shape features of pre-niche simulations.

## Bull and Byrne model

**Fig. 2:**
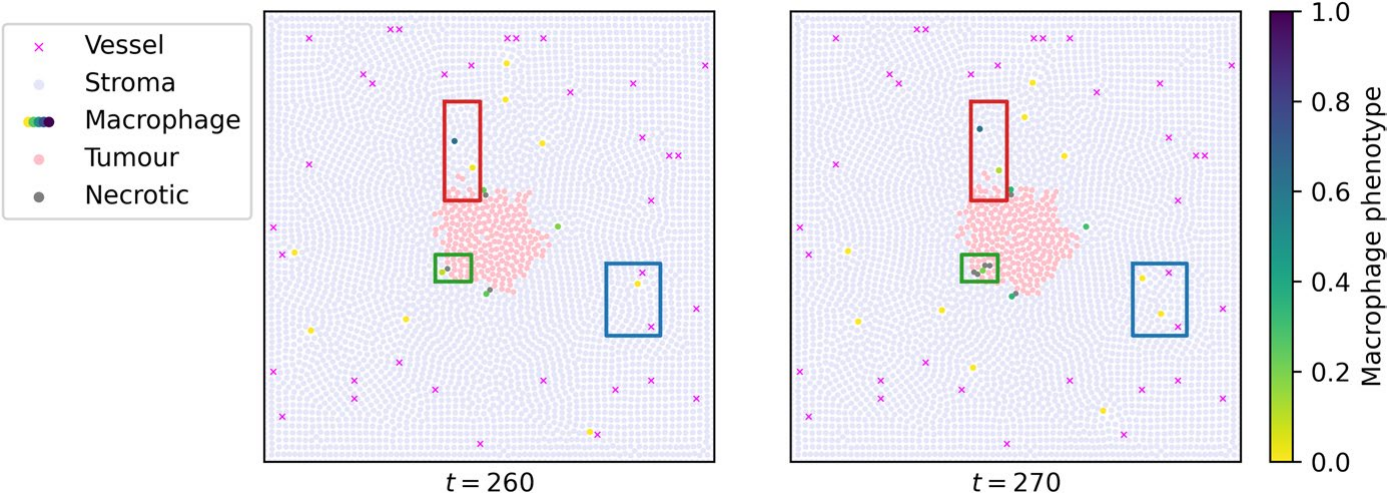
Snapshots of an ABM simulation illustrating how macrophage behaviour changes with phenotype. M_1_-like macrophages have phenotype values closer to 0 (yellow/green), whereas M_2_-like macrophages have phenotype values closer to 1 (blue). Blue box (right): M_1_-like macrophages extravasate from blood vessels and migrate towards the tumour. Green box (centre): An M_1_-like macrophage infiltrates the tumour and kills tumour cells, turning them into necrotic cells. Red box (top): Tumour cells proliferate; an M_1_-like macrophage adopts a more M_2_-like phenotype after being exposed to TGF-$\beta$ near the tumour; an M_2_-like macrophage moves towards blood vessels, attracting two tumour cells with it

We briefly describe the two-dimensional, off-lattice agent-based model (ABM) of tumour-immune interactions developed by Bull and Byrne (2023) (see Figs. 1 and  2). The model tracks macrophages, stromal, tumour and necrotic cells, including their spatial coordinates and effect on the environment, as well as ambient levels of several diffusible species, including oxygen which is supplied by blood vessels at fixed points.

Each macrophage is associated with a subcellular variable or *phenotype* $p \in [0,1]$ which governs its behavior: anti-tumour macrophages, also called M_1_, have low *p* values, *extravasate* from blood vessels, migrate toward tumour cells (up spatial gradients of the diffusible species CSF-1), and attempt to kill them on contact. Exposure to TGF-$\beta$, near the tumour, causes macrophages to transition to an M_2_, or pro-tumour phenotype, which is associated with high values of *p* ($p \ge 0.5$). Pro-tumour macrophages migrate towards blood vessels (up spatial gradients of the diffusible species CXCL12). Paracrine signalling between the M_2_ macrophages and the tumour cells (mediated by the diffusible species EGF), causes the tumour cells to move with the M_2_ macrophages, towards the blood vessels, where they intravasate and then metastasise to other parts of the body.

Stromal and tumour cells proliferate in response to oxygen supplied by the blood vessels. If they have sufficient oxygen, the cells proliferate, with tumour cells typically having a lower oxygen threshold for proliferation than stromal cells. If oxygen levels are too low for proliferation, or if a tumour cell is exposed to an M_1_ macrophage for too long, then it becomes necrotic. Stromal cells move primarily in response to physical forces exerted by surrounding cells, while tumour cells are also subject to chemotactic forces, which direct their movement up spatial gradients of EGF. Macrophages move in response to physical forces and explore their local environment via a random walk, as well as following chemotactic gradients of key diffusible species (CSF-1 for M_1 _and CXCL12 for M_2_ macrophages).

For further details of the ABM, and code to reproduce it within the Chaste (Cancer, Heart and Soft Tissue Environment) framework (Cooper et al. 2020), we refer the interested reader to Bull and Byrne (2023).

**Fig. 3:**
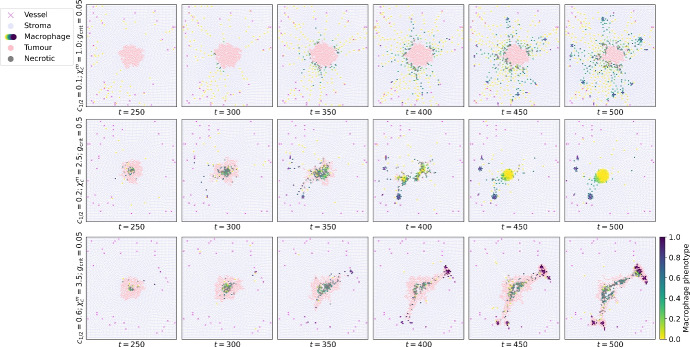
Diverse patterns of tumour growth. Series of plots showing how the spatial patterns generated by the ABM change as we vary three parameters of interest: the extravasation rate $c_{1/2}$, the chemotactic sensitivity of M_1_macrophages to CSF-1 $\chi _c^m$, and the critical TGF-$\beta$ threshold at which macrophages start to transition from an M_1 _to an M_2 _phenotype $g_\textrm{crit}$. The simulations illustrate three possible outcomes at $t = 500$. Top: Compact tumour mass in equilibrium; the dominant macrophage phenotype is M_1_. Middle: Tumour elimination; the dominant macrophage phenotype is M_1_. Bottom: Tumour escape; the dominant macrophage phenotype is M_2_

From the ABM, we generate 1485 time series of tumour cells, macrophages and blood vessel locations, spanning 0–500 hours and sampled every 10 hours. We vary three parameters that regulate the behaviour of the macrophages: the extravasation rate $c_{1/2}$, the chemotactic sensitivity $\chi _c^m$ to spatial gradients in CSF-1, and the critical threshold $g_\textrm{crit}$ of TGF-$\beta$ exposure levels above which a macrophage’s phenotype $p_i$ increases at a constant rate. Fig. 3 illustrates the range of tumour growth patterns that the ABM exhibits. We observe that the M_1_/M_2_ ratio correlates with the qualitative outcome; this is also consistent with clinical evidence in colorectal and ovarian cancers (Yang et al. 2019; Zhang et al. 2014).

We characterise a *perivascular niche* as a blood vessel with at least 10 tumour cells within 5 units of distance. This threshold was chosen to align the size of the perivascular niche area with definitions such as Hatlen and Rajagopalan (2023); Keren et al. (2018), and to select a minimum number of cells which does not arise from rapid extravasation and individual M_2_-like macrophages returning to the blood vessel.

Perivascular niches greatly increase the likelihood of metastasis, as the tumour cells can easily enter the blood vessels and be transported to other parts of the body. With this definition, perivascular niches are present in 588 (39.6%) of the simulations at $t=500$.

## Topological data analysis

**Fig. 4:**
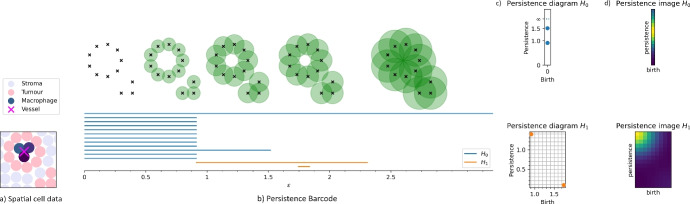
Pipeline for the Vietoris-Rips PH computation. a) Spatial coordinates of cells from simulation. b) (top) Neighbourhoods of increasing radius around tumour coordinates *T*. (bottom) The barcode $\textrm{PH}_0(K_\varepsilon )$, blue, has a bar for each component of $K_{\varepsilon _t}$ spanning the interval on which it can be seen in the filtration $K_\varepsilon$; $\textrm{PH}_1(K_\varepsilon )$, orange, contains a bar for each hole in $K_\varepsilon$ over its corresponding interval. c) The left endpoint and length (called the “birth" and “persistence", resp.) of each bar are plotted in $\mathbb {R}^2$ to form the persistence diagrams. d) A Gaussian kernel, weighted by the persistence value, is placed over every point in the diagram to form the corresponding persistence images $\textrm{PI}^{\textrm{VR}}_0(T)$ and $\textrm{PI}^{\textrm{VR}}_1(T)$

**Fig. 5:**
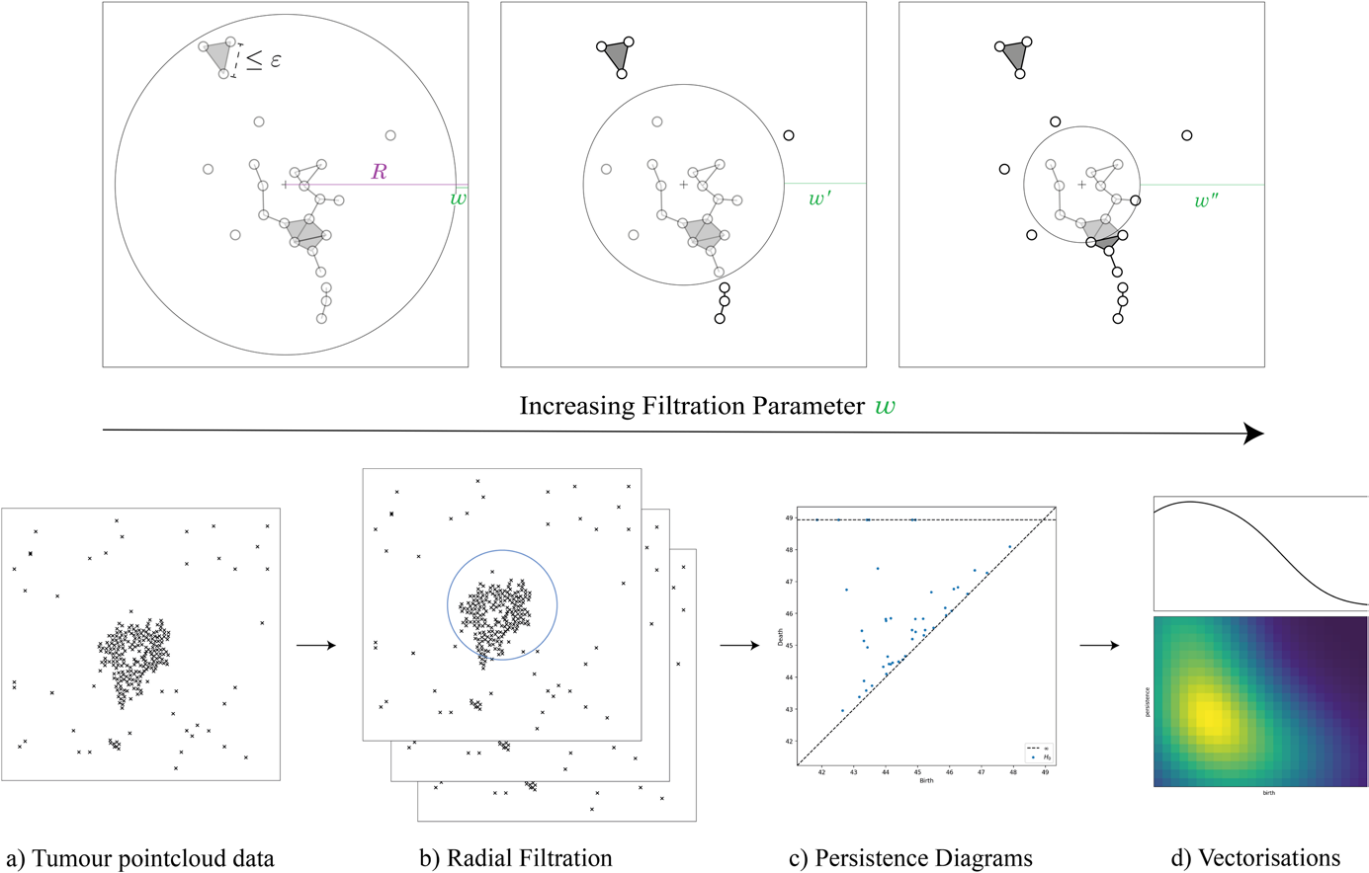
Pipeline for the radial PH computation for tumour cells. a: Input is a point cloud of cells, and basepoint $\mu$ in the centre of the simulation. b, shown in more detail at top: A simplicial complex is built by adding a point for each tumour cell, connecting neighbouring cells whenever two tumour cells have distance at most $\varepsilon$, and adding higher simplices whenever their outline is included. This complex is then filtered by radius, including the furthest points at low filtration parameters and more central points as the parameter increases. c: The persistent homology of the filtration is computed. d: The resulting diagram is converted to a two-part persistence image; a 2D image representing finite features and a 1D function representing the connected components of the full complex and their distance from the $\mu$

**Fig. 6:**
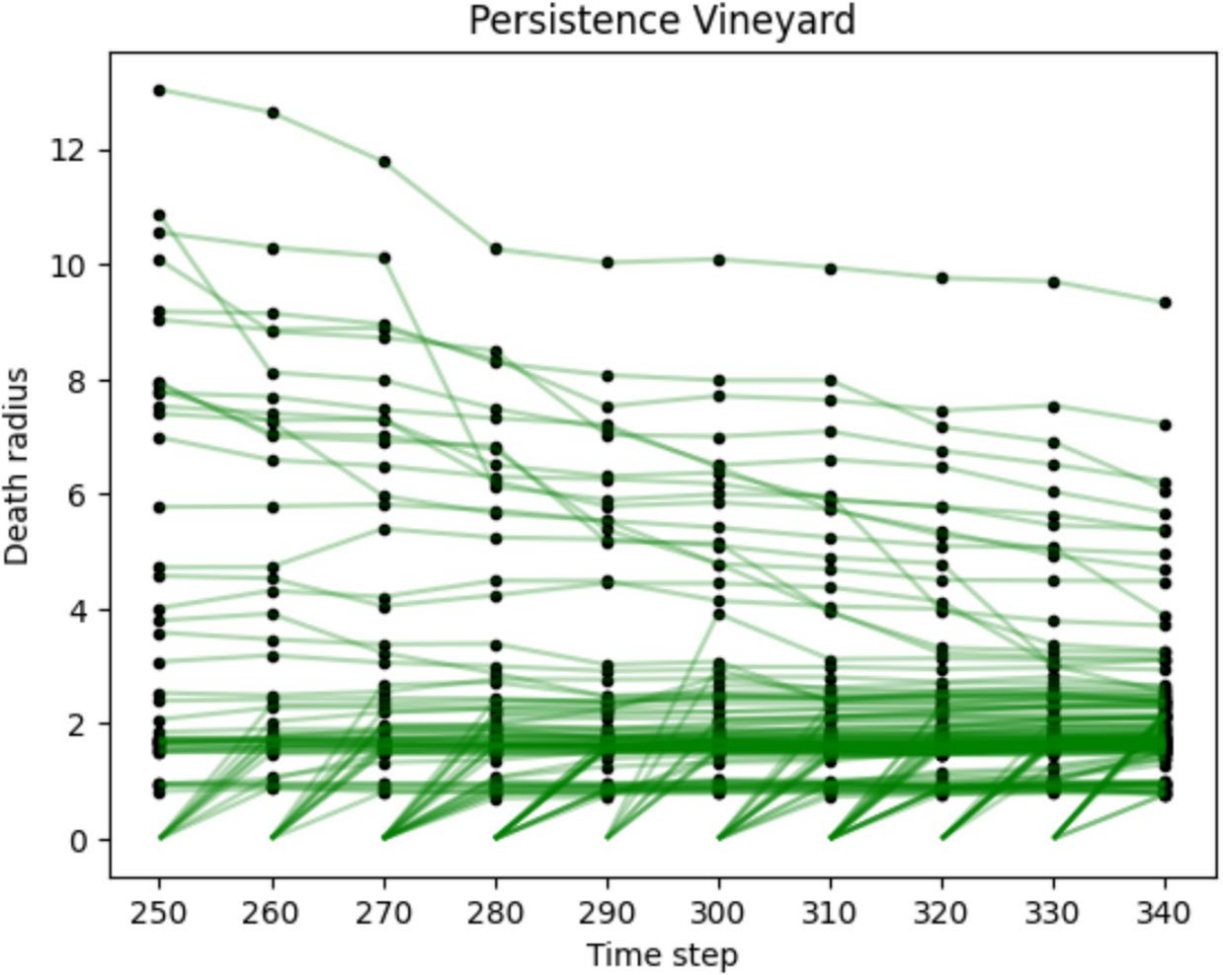
An example of a persistence vineyard approximation in dimension 0, with each vine (in green) tracking the change in death radius over time of a single feature. Each feature is generated by a single cell. At radius 0, each cell is its own feature. As the radius grows, a feature develops as other cells cluster around it, and die as the group joins a larger cluster. Here the diagrams are matched directly to illustrate continuity - this step is omitted in our methods

**Fig. 7:**
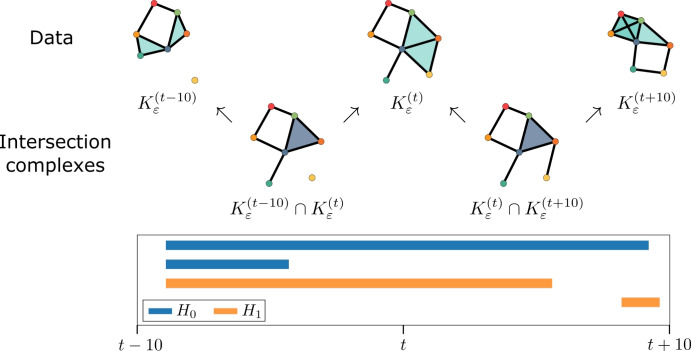
Zigzag PH detects persistent cycles across a sequence of simplicial complexes and their pairwise intersections. Top: Given a sequence of simplicial complexes, say $K^{(t-10)}_{\varepsilon }$, $K^{(t)}_{\varepsilon }$, and $K^{(t+10)}_{\varepsilon }$, zigzag PH first constructs intersected complexes for each consecutive pair, resulting in a “zigzag” of inclusion maps. Bottom: The barcode associated with the zigzag filtration

We now present the four TDA pipelines (Figs. 4, 5, 6 and 7), placing emphasis on intuition and interpretation. For a comprehensive introduction to the theory of persistent homology, see Carlsson (2009) or Ghrist (2008); we refer additionally to Adams et al. (2017) for background on the persistence image vectorisation. We first briefly describe the general TDA pipeline and then overview the four methods applied to this synthetic ABM data set.

We approximate the topology of data by first building a simplicial complex. A *simplicial complex* is a higher-dimensional generalisation of a network consisting of nodes and edges, but also triangles, tetrahedra, and higher-dimensional analogues of triangular solids. A *filtration* of a complex *K* is a function $f:K\rightarrow \mathbb {R}$ such that each sub-level set $K_c := \{\sigma \in K : f(\sigma ) \le c\}$ is itself a simplicial complex. For each sequence of discrete values $c_1\le c_2\le \dots ,\le c_n$ we obtain a sequence of inclusion maps $K_{c_1}\subseteq K_{c_2}\subseteq \dots K_{c_n}.$

The simplicial homology $H_i(K)$ of a complex *K* is an algebraic object encoding topological features of dimension *i*. The 0-dimensional homology $H_0$ is generated by connected components of the complex, and the 1-dimensional homology $H_1$ is generated by holes or loops. Persistent homology $\textrm{PH}_i$ simultaneously computes the *i*-homology of an entire filtration, and encodes how *i*-dimensional features are created or destroyed over the course of the filtration: in $K_{c_b}$, a new connected component or cluster might arise $(i=0)$, or a new hollow cycle of edges might form $(i=1)$; in $K_{c_d}$, two connected components might merge into one, or a cycle might become triangulated and filled in. The pair $(c_b, c_d)$ records the *birth-death* values of the feature with respect to the filtration, and its *persistence* is the lifespan $c_d - c_b$. By the classic Structure Theorem, $\textrm{PH}_i(K_\varepsilon )$ has a unique decomposition into intervals, which can be plotted as a barcode, or their endpoints plotted in $\mathbb {R}^2$ as a *persistence diagram*. In turn, the diagram can be vectorised into a *persistence image* (Adams et al. 2017) by placing a Gaussian kernel over all points.

### Vietoris-Rips filtration

This pipeline is summarised in Fig. 4. For a finite point cloud $X {\mathop {\subseteq }\limits ^{\text {fin}}} \mathbb {R}^2$, and radius parameter $\varepsilon \ge 0$, the *Vietoris-Rips complex* $K_\varepsilon$ associated to *X* is given by:

- a node (0-simplex) for each element $x\in X$;
- an edge (1-simplex) $\{x, y\}$ when $x, y \in X$ satisfy $d(x, y) \le \varepsilon$;
- a triangle (2-simplex) $\{x, y, z\}$ if $x, y, z \in X$ satisfy $d(x, y), d(y, z), d(z, x) \le \varepsilon$;
- more generally, an *n*-simplex $\{x_0, x_1, \dots , x_n\}$ when $d(x_i,x_j)\le \varepsilon$ for all $x_i,x_j$.

We note that although our data lies in the real 2-dimensional plane, $K_\varepsilon$ is a discrete combinatorial object which can contain components of any dimension up to $|X|-1$.

Given a finite sequence of radii $\varepsilon _0 \le \varepsilon _1 \le \dots \le \varepsilon _n$, we obtain a nested sequence of simplicial complexes $K_{\varepsilon _0} \subseteq K_{\varepsilon _1} \subseteq \dots \subseteq K_{\varepsilon _n},$ since simplices are added monotonically as $\varepsilon$ grows. This is the *Vietoris-Rips* filtration. The birth and death of each homological feature indicates its scale: at small $\varepsilon$, only neighbouring points are connected by simplices, while at large $\varepsilon$, more global features appear.

We will denote the output image by $\textrm{PI}^{\textrm{VR}}_i(X)$, where *i* is the dimension and *X* is a static point cloud of macrophages *M* or tumour cells *T* at a specific time step. We use Ripser (Bauer 2021) to compute $\textrm{PH}_i(K_\varepsilon )$ for $i=0,1$, and Persim to generate the images $\textrm{PI}^{\textrm{VR}}_0$ and $\textrm{PI}^{\textrm{VR}}_1$ from these outputs; we use the default linear weighting by persistence for Gaussian intensity.

### Radial filtration

For a finite point cloud $X {\mathop {\subseteq }\limits ^{\text {fin}}} \mathbb {R}^2$, we fix a linkage distance $\varepsilon$ and construct a (Vietoris-Rips) simplicial complex $K_{\varepsilon }$ on the point locations. This is used as the underlying complex for the filtration. As shown in Fig. 5, at parameter $w \ge 0$, the simplicial complex $K_\varepsilon ^{(w)}$ is defined

$$K_{\varepsilon }^{(w)}: = \left\{ \sigma \in K_\varepsilon \hspace{0.5em}:\hspace{0.5em} R-||x-\mu || \le w \hspace{1em}\forall x\in \sigma \right\} ,$$

where $\mu$ is a fixed basepoint in the middle of the point cloud, $||\cdot ||$ is the standard Euclidean norm, and *R* is a maximum radius such that $||\mu -x||< R$ for all points $x\in X$. In this way, $K_{\varepsilon }^{(w)}$ includes the points which are over $(R-w)$ away from the base point as 0-simplices, as well as all edges, triangles, and higher simplices between these points. Given $0 = w_0 \le w_1 \le \dots \le w_n = R$, we obtain a filtration

$$\emptyset = K_{\varepsilon }^{(w_0)} \subseteq K_{\varepsilon }^{(w_1)} \subseteq \dots \subseteq K_{\varepsilon }^{(w_n)} = K_\varepsilon$$

which we call the *radial filtration*.

We use GUDHI to construct this filtration and to compute its PH, and Persim to compute persistence images, as before. However, standard persistence images exclude features of *infinite* persistence: these are homological features that are still present at the final filtration step $K_\varepsilon$, and therefore are said to “never die". We reintroduce these features, which are parametrised in one dimension by their birth radius.

The infinite features in $H_0$ correspond to the connected components of the underlying simplicial complex $K_{\varepsilon }$. The infinite features are vectorised as a real-valued function over the range of radii by taking a sum of 1D Gaussian kernels centred at their birth times. This 1D persistence image is appended as a vector to the persistence image obtained from the finite points. The resulting image is called $\textrm{PI}^\textrm{rad}$.

### Time-dependent topological analyses

We now turn to the first of two time-dependent analyses. Given a finite point cloud $X_t {\mathop {\subseteq }\limits ^{\text {fin}}} \mathbb {R}^2$, that is continuously changing location over time, we track how and when clusters form, persist and die. Rather than study a single time snapshot, we use the cumulative trajectory from many timesteps.

#### Persistence vineyard

A seemingly naïve method of tracking evolution over time is to compute $\operatorname {PH}_i(K^t_\epsilon )$, where $K^t_\varepsilon$ is the Vietoris-Rips filtration associated to $X_t$, for each individual time step *t*. The Vietoris-Rips stability theorem Chazal et al. (2009) states that a small perturbation of the input point cloud (in Gromov-Hausdorff distance) only elicits a small change in the resultant persistence diagram. If the time series is continuous with respect to this distance, then, it follows that points in the persistence diagram far from the diagonal vary continuously in *t*.

When the persistence diagrams are stacked serially, with time as a third axis, in addition to birth and death, the points in the persistence diagrams trace out smooth curves (or when discrete time steps are taken, a piecewise-linear approximation) called *vines* (see Fig. 6).

Persistence vineyards do not admit an obvious vectorisation, but we can encode the stacked persistence diagrams in the persistence image by plotting the aligned Rips diagrams and allowing the Gaussian kernel to spread across layers. As any point $(\varepsilon _b, \varepsilon _d)$ in $\operatorname {PH}_0$ of the filtration $K_{\varepsilon }^{(t)}$ at time step *t* is born at $\varepsilon _b = 0$, the *vineyard* of vines lies entirely in the two-dimensional plane $\varepsilon _b=0$. Therefore, we do not explicitly extract the vines; rather, by abusing notation, we can consider $(t, \varepsilon _d)$ as a birth-persistence pair and thus obtain a persistence image. We discard the single point $(\varepsilon _b, \varepsilon _d) = (0, \infty )$ at all time steps since it does not contribute additional information in the Rips filtration.

Representing the corresponding stacked persistence diagram in dimension 0 with a persistence heat-kernel image as in previous sections, we denote the result $\textrm{PI}^{\textrm{vin}}_0(M)$.

#### Zigzag persistence

One main drawback of the persistence vineyard is that it typically identifies features across consecutive time steps based on an imposed matching of topological features, where we assume that features (*b*, *d*) and $(b',d')$ belonging to consecutive time steps represent the evolution of the same feature if they have a similar birth and death time relative to other features. If the time steps are sufficiently dense, this may be accurate, but in general, this assumption does not hold. Ideally, we would like to incorporate knowledge of which cells are co-located in a topological feature to determine if a feature persists from one time step to the next, or if it dies between time steps while a similar-looking feature is born. We can accomplish this via a *zigzag *filtration (Fig. 7).

In order to track the actual, rather than estimated, persistence over time of homological features, we implement a non-monotonic filtration called a *zigzag* (Carlsson and De Silva 2010). Zigzag filtrations exploit simplicial maps to compute the persistent homology of a sequence of simplicial complexes which is not strictly growing, but can both add and remove simplices. This allows us to compute persistence over time and capture the topological dynamics. However, it requires some pre-processing: we must alter our sequence so that each map is either an inclusion or a restriction. To do this, we construct the *intersection complex* $K_\varepsilon ^t\cap K^{t+10}_\varepsilon$, and compute the persistent homology of the sequence as shown in Fig. 7, using the natural inclusion maps of the intersection. This produces the *zigzag filtration*

$$\begin{aligned} \cdots \rightarrow K^{(t - 10)}_{\varepsilon } \leftarrow K^{(t - 10)}_{\varepsilon } \cap K^{(t)}_{\varepsilon } \rightarrow K^{(t)}_{\varepsilon } \leftarrow K^{(t)}_{\varepsilon } \cap K^{(t + 10)}_{\varepsilon } \rightarrow K^{(t+10)}_{\varepsilon } \leftarrow \cdots . \end{aligned}$$

The corresponding zigzag persistence module

$$\begin{aligned} \cdots \rightarrow PH_i(K^{(t - 10)}_{\varepsilon }) \leftarrow PH_i(K^{(t - 10)}_{\varepsilon } \cap K^{(t)}_{\varepsilon }) \rightarrow PH_i(K^{(t)}_{\varepsilon }) \leftarrow PH_i(K^{(t)}_{\varepsilon } \cap K^{(t + 10)}_{\varepsilon }) \rightarrow PH_i(K^{(t+10)}_{\varepsilon }) \leftarrow \cdots \end{aligned}$$

enjoys the same unique decomposition (Botnan 2015) and stability (Botnan and Lesnick 2018) properties as ordinary PH for all *i*. As a result, we can compute a *zigzag barcode* for the zigzag persistence module (see Fig. 7). We vectorise the persistent homology of the zigzag filtration with a persistence image, in exactly the same manner as Sect. 3.1. We call the result $\textrm{PI}^{\textrm{zz}}_0(M)$, as we use the 0th homology of the macrophage point cloud in our analysis.

#### Comparing vineyards and zigzags

Lastly, we highlight the different information provided by vineyards and zigzags. As previously discussed, in the vineyard, points in the persistence diagram represent multi-scale features connected by topological similarity over time. In the zigzag, a scale of neighbour connection must be fixed, similar to the radial filtration, and each simplex is tracked over time. This leads to slightly different dynamics, as the vineyard may detect spatial continuity that the zigzag does not, and the zigzag may detect temporal continuity that the vineyard does not.

### Classification

We use a cross-validated logistic regression classifier to evaluate membership into two classes of tumour: those that will go on to form perivascular niches by time 500, and those that do not. In this analysis we use the sklearn Python package with default cross-validation parameters, i.e. stratified 5-fold cross-validation repeated 10 times.

Importantly, logistic regression enables us to perform inverse analysis; using the regression coefficients, we can identify those regions of the persistence image which are most indicative of class membership. For example, high contrast at low persistence values in $\textrm{PI}^{\textrm{VR}}_0$ indicates that the number of cell clusters at low single-linkage radii differs significantly between the groups, and the high contrast along the diagonal of the zigzag filtration regression coefficients indicates that features born at any time and lasting through to the final time step are most significant.

**Benchmarks.** In addition to the topological methods, we also implemented classifiers trained on four benchmark statistics: tumour cell count, macrophage count, macrophage phenotype ratio M_1_/M_2_, and minimum tumour-vessel distance. We selected these statistics to provide a competitive benchmark relating our work to other methods of classifying outcome from cell location and phenotype data.

Tumour cell count is a straightforward measure of tumour size, a traditional prognostic factor in cancer biology. Tumour cell count will naturally be much lower in tumour elimination cases, giving this measurement an edge in detecting elimination behaviour. Macrophage cell counts, while less directly tied to outcome, are typically found to have considerable prognostic significance as well (Cortese et al. 2020).

Macrophage phenotype ratio is the most sophisticated of the four benchmarks, as phenotype is more difficult to detect and quantify than cell location. In real data, various markers for phenotype are used to classify macrophages into M_1_ and M_2_ classes (Eftimie 2020), and the resulting ratio M_1_/M_2_ is a widely-used measure of macrophage polarisation (see e.g. Zhang et al. 2014). In our model, phenotype is a simulated dynamic variable associated to each immune cell; we use the continuous phenotype ratio values and let the classifier set the decision boundary optimally.

Tumour-vessel distance should naturally perform very well, given our definition of a perivascular niche (i.e. the presence of at least 10 tumour cells in close proximity to a blood vessel). High accuracy at time step 500 is therefore almost a certainty, with tumour-vessel distance at late time-steps expected to correlate accordingly.

### Computational complexity

In our analysis, the highest computational cost by far was the agent-based model. However, there is some variance in computation time for classification among the methods.

Classic persistent homology algorithms (via boundary matrix reduction) are $O(n^3)$ where *n* is the number of simplices. Depending on the filtration, *n* in a standard Rips filtration can be up to $O(c^3)$ (*c* the number of cells) for dimension-1 homology. In practice, however, it is often much less, especially using state-of-the-art reductions. This can be improved to $O(n\log (n))$ with $n\sim O(c^2)$ for 0-dimensional homology. Ripser uses several additional computational speed-ups, including sparsification, implicit representations of boundary operators, and reductions from discrete Morse theory (Bauer 2021). While it has the same worst case complexity, in practice it tends to require much lighter space and compute time. We note also that the number of simplices is different for each of our filtrations, and that the size of the resulting persistence image (which depends only on dimension and the *resolution* specified in persim) will affect training time for the classifier.

- Vietoris-Rips: computed using Ripser, on average much faster than the worst case run time $\sim c^9$ for dimension 1 and $c^2\log (c)$ for dimension 0.
- Radial: the fastest of the methods. It uses Ripser in dimension zero, with a lightweight complex size of $n\sim c$ for $\varepsilon \le 1$, as we use, resulting in $O(c\log (c))$ run time.
- Vineyards: As the images are stacked 0-d Vietoris-Rips images, compute time increases by a factor of 10, but still on the order of $c^2\log (c)$.
- Zigzag: Despite having a lightweight filtration, as the zigzag is not monotonic, the software currently available to compute PH is less efficient. We used Gudhi to build the complex as described in Sect. 3.3.2 explicitly. Experimentally, this construction is currently the slowest at $\sim c^4$, but may be comparable to the others when the known speed-ups are adapted to non-monotonic filtrations.

## Results and discussion

We apply the TDA methods as described in Sect. 3 to analyse patterns of tumour and macrophage cell locations:

- 4.1 Vietoris-Rips persistence images $\textrm{PI}^{\textrm{VR}}_0(T),\textrm{PI}^{\textrm{VR}}_1(T),\textrm{PI}^{\textrm{VR}}_0(M),\textrm{PI}^{\textrm{VR}}_1(M)$ of tumour and macrophage cell locations in dimensions 0 and 1;
- 4.2 radial persistence images $\textrm{PI}^{\textrm{rad}}_0(T)$ of tumour cells in dim. 0;
- 4.3.1 persistence vineyard images $\textrm{PI}^{\textrm{vin}}_0(M)$ for macrophages in dim. 0; and
- 4.3.2 zigzag persistence images $\textrm{PI}^{\textrm{zz}}_0(M)$ of macrophages dim. 0.

The first two, $\textrm{PI}^{\textrm{VR}}_i$ and $\textrm{PI}^{\textrm{rad}}_0(T)$, are static data analysis methods at time *t*, while $\textrm{PI}^{\textrm{zz}}_0(M)$ and $\textrm{PI}^{\textrm{vin}}_0(M)$ analyse dynamics over a time period preceding *t*. The dimension 0 images track connected components over different positions, scales, or time. The dimension 1 images record the scale of holes in the cell clusters.

We compare these methods against benchmarks using a dataset simulated from the Bull and Byrne agent-based model. For a parameter sweep of extravasation rate $c_{1/2}$ (9 increments), chemotactic sensitivity $\chi _c^m$ (9 increments), and critical TGF-$\beta$ threshold $g_{\text{crit}}$ (2 options), we generated 10 models runs for each of the 162 parameter combinations. Due to limited high-performance compute resources, there were a total of 1485 successful model runs, each spanning 0-500 hours (see Fig. 3). Each timestep of each run produces a list of cell details: the cell index, type (tumour, macrophage, or blood vessel), and off-lattice (*x*, *y*)-coordinates within the $[0,50]\times [0,50]$ square in $\mathbb {R}^2.$

Although simulations are generated with high temporal resolution, real data is often much more limited. In our use of static methods, we analyse single timesteps at 250, 300, 350, 400, 450, and 500 hours. For dynamic methods, we analyse 100-hour windows in 10-hour increments with the same end times at 250, 300, 350, 400, 450, and 500 hours, e.g. 150:10:240 for timestep 250.

**Fig. 8:**
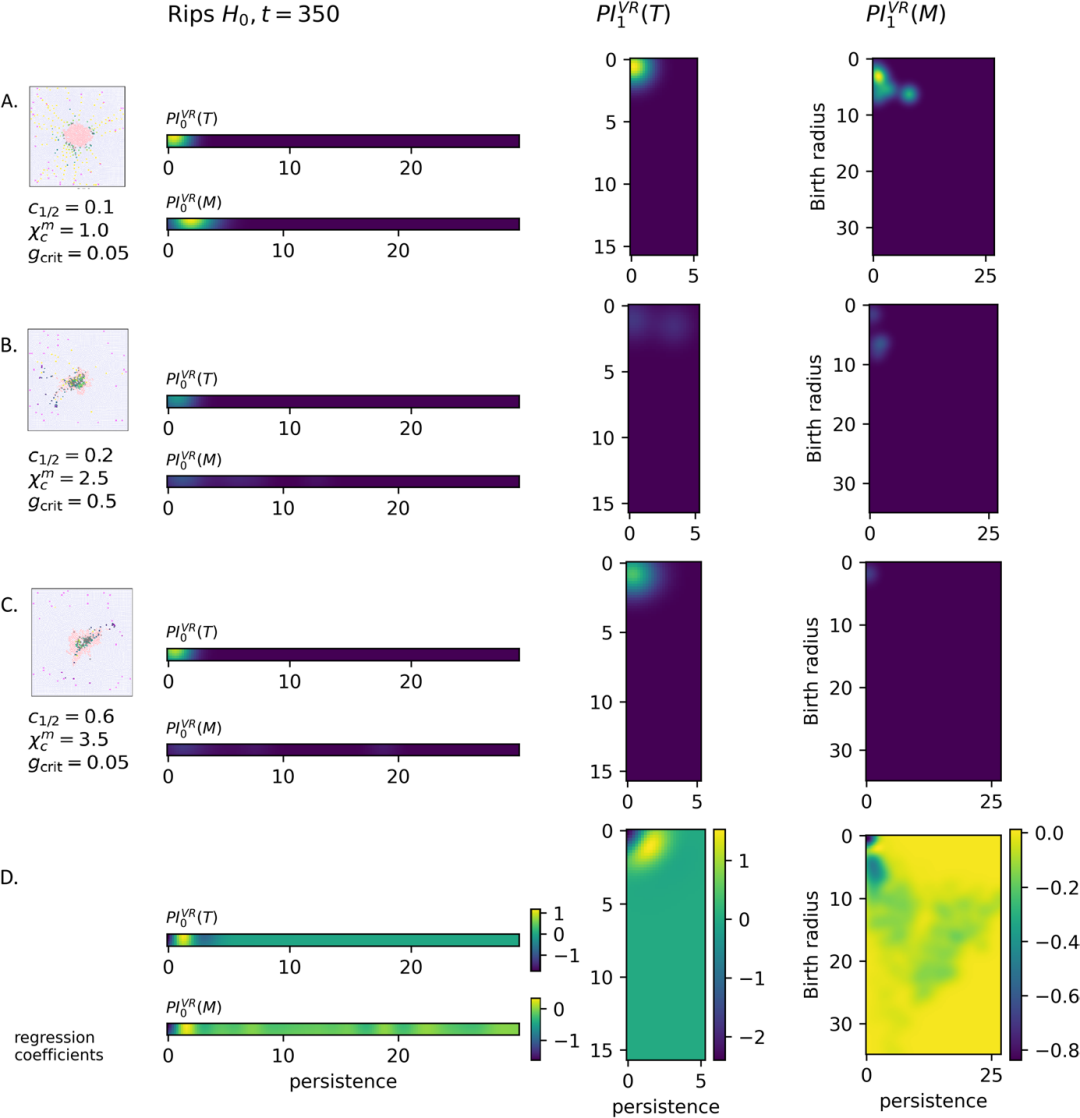
Persistent images of VR filtrations. A, B, C: Sample persistence images at time $t = 350$ for equilibrium, elimination, and escape classes (resp.) as shown in Fig. 3, originating from varying extravasation rate $c_{1/2}$, chemotactic sensitivity $\chi ^m_c$, and critical TGF-$\beta$ threshold $g_{\text{ crit }}$. Each of the sample simulations has persistence images $\textrm{PI}^{\textrm{VR}}_0(T)$, $\textrm{PI}^{\textrm{VR}}_1(T)$, $\textrm{PI}^{\textrm{VR}}_0(M)$, and $\textrm{PI}^{\textrm{VR}}_1(M)$. D: Regression coefficients identify regions of the persistence image that are most important for distinguishing the classes

We assign a class label of 1 to time series that exhibit at least one perivascular niche at time 500 h, and assign 0 otherwise. For each persistence image, we train a logistic regression classifier as described in Sect. 3.4 and determine its ability to predict whether a given simulation time series will go on to form a perivascular niche. In Sects. 4.1, 4.2, and 4.3, corresponding to Figs. 8, 10, and 11, respectively, we examine the logistic regression coefficients and overall class differences between the images. This allows us to identify and interpret the regions of the persistence images that are most strongly linked (positively or negatively) to the future development of perivascular niches, and to determine what topological features they represent. In Sect. 4.4, we present the accuracy scores given by the classifier at different time steps for each method and compare the performance of topological summaries with classic heuristics and with each other.

### Vietoris-Rips persistence identifies tumour clusters and immune infiltration

Vietoris-Rips persistence is used to analyse the pairwise distances between point clouds of tumour cells *T* and point clouds of macrophages *M*. We compute persistent homology of the Vietoris-Rips filtration in dimensions 0 and 1, take the persistence images of the homological features, and denote the result $\textrm{PI}^{\textrm{VR}}_i(X),$ where $i\in \{0,1\}$ is the homological dimension and $X \in \{M,T\}$ is the cell type. $\textrm{PI}^{\textrm{VR}}_0$ provides a multi-scale measure of the number, size, and proximity of the clusters of a particular cell type, whereas $\textrm{PI}^{\textrm{VR}}_1$ measures the number, scale, and enclosure size of cavities inside those cell clusters. The logistic regression coefficients and sample persistence images from the Vietoris-Rips complexes are shown in Fig.  8.

#### Vietoris-Rips results 

**Tumour components –**$\varvec{\textrm{PI}^{\textrm{VR}}_0(T)}$. For tumour cells, $\textrm{PI}^{\textrm{VR}}_0(T)$ tracks the clusters of tumour cells and their proximity. Unless the tumour is separated into multiple distinct components, $\textrm{PI}^{\textrm{VR}}_0(T)$ will have persistence only slightly larger than the cell radius 0.5. A feature of larger persistence *p* indicates a separate component at distance *p* from the rest of the tumour.

The logistic regression coefficients give some insight into the differences between the niche and control cases. From Fig. 8, we can see that the hallmark of the niche classes is components of persistence $1-2$, with non-niche cases showing more features of both smaller (0-1) and larger (2-4) persistence.

**Fig. 9:**
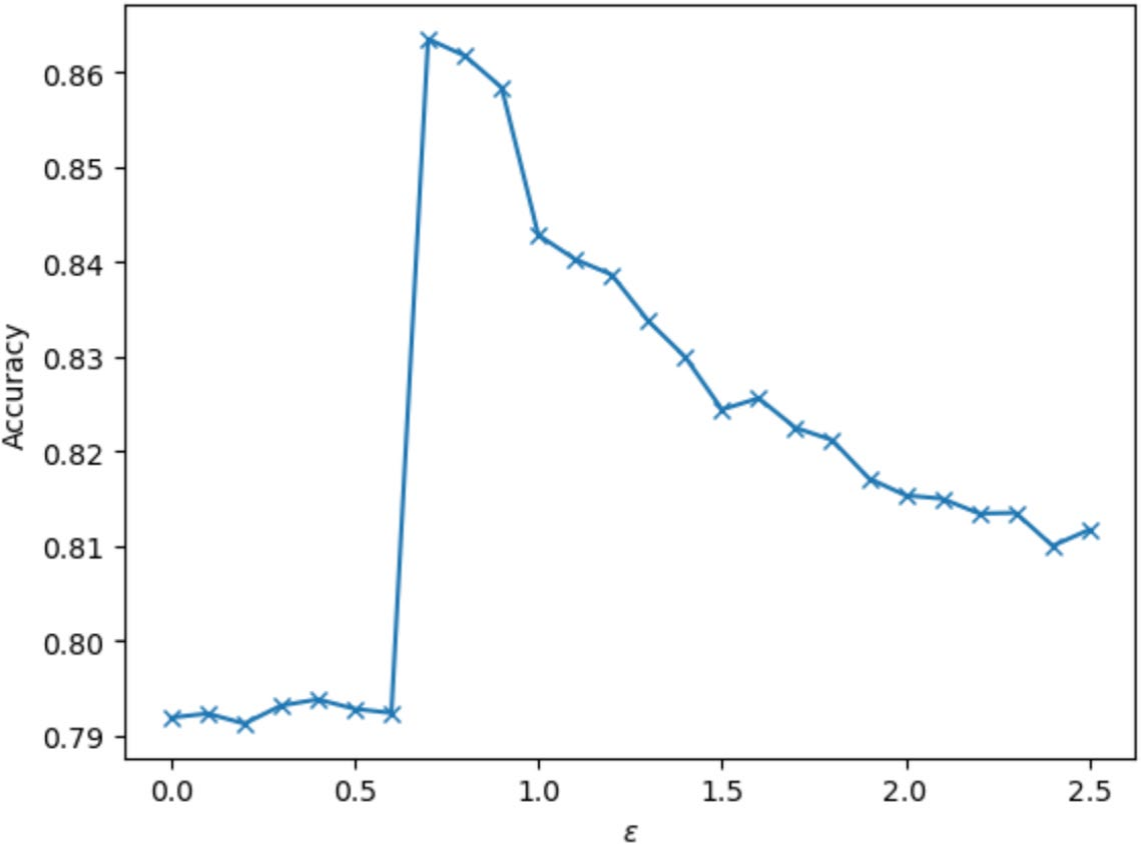
A coarse $\epsilon$-sweep to find the optimal connection radius (0.8) of tumour component, where 1.0 is the diameter of each cell in the simulation. This gives some additional insight into the proximity of tumour cells and what constitutes a significant distance

The smaller features are likely individual tumour cells, which die around the optimal linking radius of 0.7 found in the $\epsilon$-sweep for the radial filtration (See Fig. 9). The features of larger persistence are disconnected from the body of the tumour, with at least one non-tumour cell in between. This can occur during elimination, or in the process of migration toward a blood vessel, although the results indicate that disconnection is more characteristic of non-niche cases.

The logistic regression classifier trained on $\textrm{PI}^{\textrm{VR}}_0(T)$ outperforms the classifier trained on tumour cell count, which shows that the number and relative distance of tumour components differs meaningfully between the classes. However, $\textrm{PI}^{\textrm{VR}}_0(T)$ does not predict perivascular niches as well as other topological methods (see Fig. 12). While fragmentation of the tumours occurs in elimination cases and equilibrium cases, it may also occur during the process of escape.

**Tumour holes –**$\varvec{\textrm{PI}^{\textrm{VR}}_1(T)}$. $\textrm{PI}^{\textrm{VR}}_1(T)$, which represents cavities inside tumours, shows improved performance of the classifier. The logistic regression coefficients (see Fig. 8) contain a dark spot at (0, 0) which is associated with the non-niche class. These are cycles of tumour cells with very small spacing in between and a small loop radius so that the interior is only one or two cells wide. The bright yellow spot near (1.5, 1.5), which is correlated with the niche cases, represent loops of tumour cells that have a linking radius of 0-2 cells in between and an interior radius around two cells wide, or four cells in diameter. This pattern can be seen clearly in the examples shown, with a larger and more pronounced tumour hole in the escape case, a looser tumour loop in the elimination case, and a high number of loops of very small persistence in the equilibrium case.

While the coefficients do not tell us which other types of cells are in the interior of the cavity, an inspection of the simulations shows tumour loops around macrophages and around a single blood vessel. Both scenarios are associated primarily and frequently with simulations that give rise to escape, which we hypothesise drives the performance of classifiers using $\textrm{PI}^{\textrm{VR}}_1(T)$ as input. The results also suggest that the number of infiltrated macrophages may play an important role in differentiating between the elimination and escape case at early time steps.

**Macrophage clusters –**$\varvec{\textrm{PI}^{\textrm{VR}}_0(M)}$. Macrophages have very different homological profiles. For both training classes, macrophage clusters form at a larger radius than tumour cells, and large-persistence features have more weight in classification. However, the logistic regression coefficients of $\textrm{PI}^{\textrm{VR}}_0(M)$ show a similar pattern to $\textrm{PI}^{\textrm{VR}}_0(T)$: a positive correlation of features with persistence 1-2 in the niche case, with all other persistence values linked with lower likelihood of niche formation (see Fig. 8).

The logistic regression coefficients indicate that closely spaced, but not packed, macrophages may be a signature of the niche case, while the presence of densely-packed or diffuse clouds of macrophages predict non-niche behaviour. The larger linkage radius of macrophage components was also seen in our $\epsilon$-sweeps for the radial and zigzag filtrations. We think this is likely because macrophages move independently and do not proliferate, so they are less likely than tumour cells to form densely-packed clusters. Classifiers trained on $\textrm{PI}^{\textrm{VR}}_0(M)$ outperform those trained on macrophage phenotype ratio and macrophage count (see Fig. 12), at timesteps up to 400 h. Based on our tests, $\textrm{PI}^{\textrm{VR}}_0(M)$ predicts perivascular niche formation up to 150 h earlier than macrophage count or phenotype ratio. As the number of features in $\textrm{PI}^{\textrm{VR}}_0(M)$ coincides with the total number of macrophages, this means that the spatial clustering of macrophages encodes useful information above and beyond the macrophage count.

**Macrophage holes –** $\varvec{\textrm{PI}^{\textrm{VR}}_1(M)}$ Interestingly, as shown in Fig. 8, the presence of any feature in $\textrm{PI}^{\textrm{VR}}_1(M)$ is correlated with decreased likelihood of niche formation, at approximately the same accuracy levels as phenotype ratio M_1_/M_2_(Fig. 12).

In the example images, we see some patterns of macrophage behaviour which may be driving this result. $\textrm{PI}^{\textrm{VR}}_1(M)$ has a dominant feature in the equilibrium case, as the macrophages assemble around the perimeter of the tumour, with the tumour mass enforcing a large cavity in the macrophage point cloud. In the elimination case, tumour fragmentation can create multiple macrophage voids as each tumour fragment become surrounded by macrophages. Additionally, extravasation may cause macrophage loops surrounding blood vessels. In general, however, we expect the majority of macrophage loops to contain tumour cells in the interior.

Based on the examples and the coefficients, we may also ascribe some of these results to the differing behaviour of M_1_-like and M_2_-like macrophages, which may in part explain why the accuracy level is so similar to phenotype ratio. M_1_macrophages will remain on the boundary of the tumour, accumulating over time to form more well-defined loops around tumour components, while M_2_macrophages will return to the blood vessel.

### Radial persistence characterises tumour tortuosity

The *radial filtration*, as shown in Fig. 5, captures the tortuosity and fragmentation of the tumour. Tumour cells outside radius *r* are clustered and tracked as *r* decreases, including cells nearer and nearer to the centre, noting when clusters merge. For example, a small outgrowth on a large tumour will have a high birth radius and low persistence and appear as a spot in the lower left of the persistence image. A large outgrowth on a small tumour will have a smaller birth distance and larger persistence, and it will appear in the upper right of the persistence image.

The persistent homology $H_0$ therefore measures the variability of the outer boundary (e.g. tortuosity) of the tumour and the overall compactness of the central tumour. The infinite features of $H_0$, vectorised separately and appended as per Sect. 3.2, represent connected components in the underlying tumour complex.

**Linking radius** We choose the cell neighbour distance $\varepsilon =0.7$ based on a coarse parameter sweep, as shown in Fig. 9. This linking radius was seen to maximise the classification accuracy of the filtration.

The optimal linkage distance $\varepsilon = 0.7$ is only slightly greater than the cell radius of 0.5, indicating that tumour cells in the same component tend to be quite close together, and that tumour cells spaced more than 0.4, boundary to boundary, from the main tumour may be considered separated for the purposes of classification.

In our model, the central basepoint is aligned for all simulations at $(25,25)$, where the tumour is initially located, and we use $R=35$. In more general cases, including with real data, a fixed basepoint cannot be assumed. We propose the centre of the tumour mass as a basepoint in these cases. See Sect. 5.1 for more discussion.

**Fig. 10:**
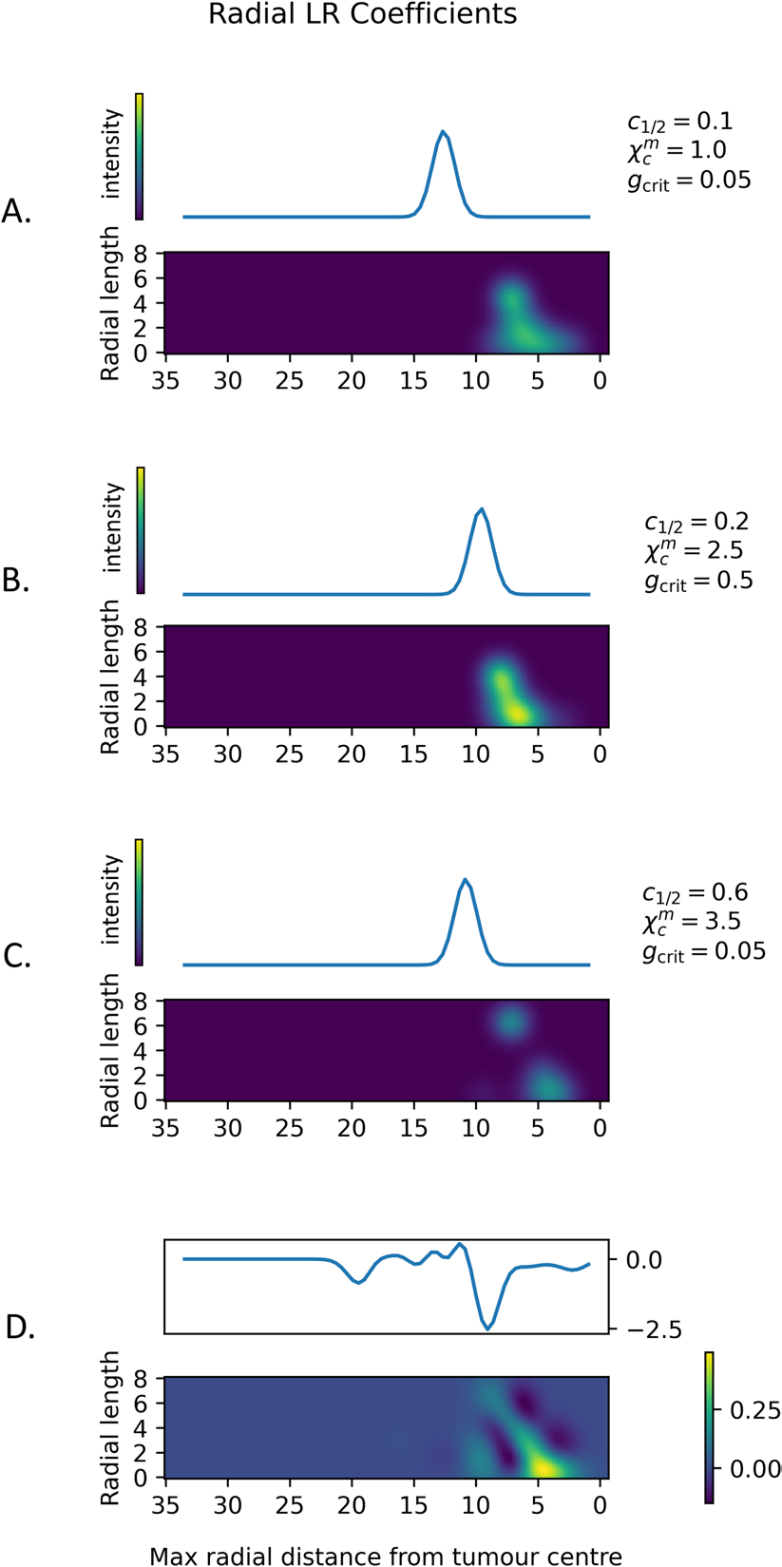
**Persistence images of radial filtrations.** A, B, C: Sample persistence images $\textrm{PI}^{\textrm{rad}}_0(T)$ for the equilibrium, elimination, and escape example cases at $t = 350$ (see Figs. 3 and 8). D: Logistic regression coefficients for the classifier. The features in the persistence image represent separate outgrowths of the tumour. For all subfigures, the *x*-axis is the radial distance at which features are born. For a single outgrowth, birth (*x*-coordinate) occurs at the furthest cell from the centre of the outgrowth, which we call the maximum radial distance of the feature. For finite features (35$\times$8 rectangle), the persistence (*y*-axis) represents the length of its protrusion from the main body of the tumour in terms of the difference in radius. The blue curve above each image records the infinite features which arise from completely disconnected components of the tumour. These have no death radii, so are represented by a vector using the same Gaussian kernal as the persistence image (plotted vertically)

**Radial components –** $\varvec{\textrm{PI}^{\textrm{rad}}_0(T)}$ The logistic regression coefficients and sample persistence images from the radial filtration are shown in Fig. 10.

The logistic regression coefficients show that infinite features are most prominent in the non-niche class, which is already seen in results from $\textrm{PI}^{\textrm{VR}}_0(T)$ at $r = \varepsilon =0.7$. What the infinite features show beyond $\textrm{PI}^{\textrm{VR}}_0(T)$ is the distribution of radii for relevant features. The logistic regression coefficients give a negative weight to almost all infinite features, but particularly those of radius around 10 and 20, with a slight positive weight to features of radius 12-15. A fragmented tumour would show a multi-modal curve of infinite features in $\textrm{PI}^{\textrm{rad}}_0(T)$ with a larger total area under the curve, so the overall negative logistic regression coefficients may be due to the prevalence of infinite features of $\textrm{PI}^{\textrm{rad}}_0(T)$. The positive regression coefficients may indicate that a main component radius around 12-15 might override other indicators.

The finite logistic regression coefficients show a similar dependence on scale. Although the positive coefficients outweigh the negative coefficients, indicating that outgrowths in general are more strongly correlated with the niche case, there are regions that show the outgrowth pattern of non-niche cases. In the niche case, a tumour of radius 5 begins showing outgrowths, the length of which increases linearly as the tumour increases in radius, up to a tumour radius of 10 with a radial length of 6-8, long enough to reach a blood vessel. A larger radius, with low persistence, is associated with the non-niche class; from inspection, likely equilibrium. There is also an association of high persistence with a smaller radius to the non-niche class. We suspect this is due to the fragmentation that occurs in the elimination case, as with the infinite features.

The three featured examples all consist of a single tumour component, so the infinite feature curve has a single mode, centred on the maximum radius of the tumour. Our example curves show the largest radius in the equilibrium case and the lowest radius in the elimination case. The finite portions of $\textrm{PI}^{\textrm{rad}}_0(T)$ reveal strong boundary tortuosity in the elimination case, with a similar, but less dramatic, pattern for the equilibrium case. The escape case has fewer outgrowths, but significantly larger persistence of outgrowths.

We note that the coefficients shown in Fig. 10 apply only to timestep 350, so the values may change as the simulation progresses. If the values do vary between timesteps, we may need relatively precise normalisation of time and distances in future applications.

### Dynamic methods

**Fig. 11:**
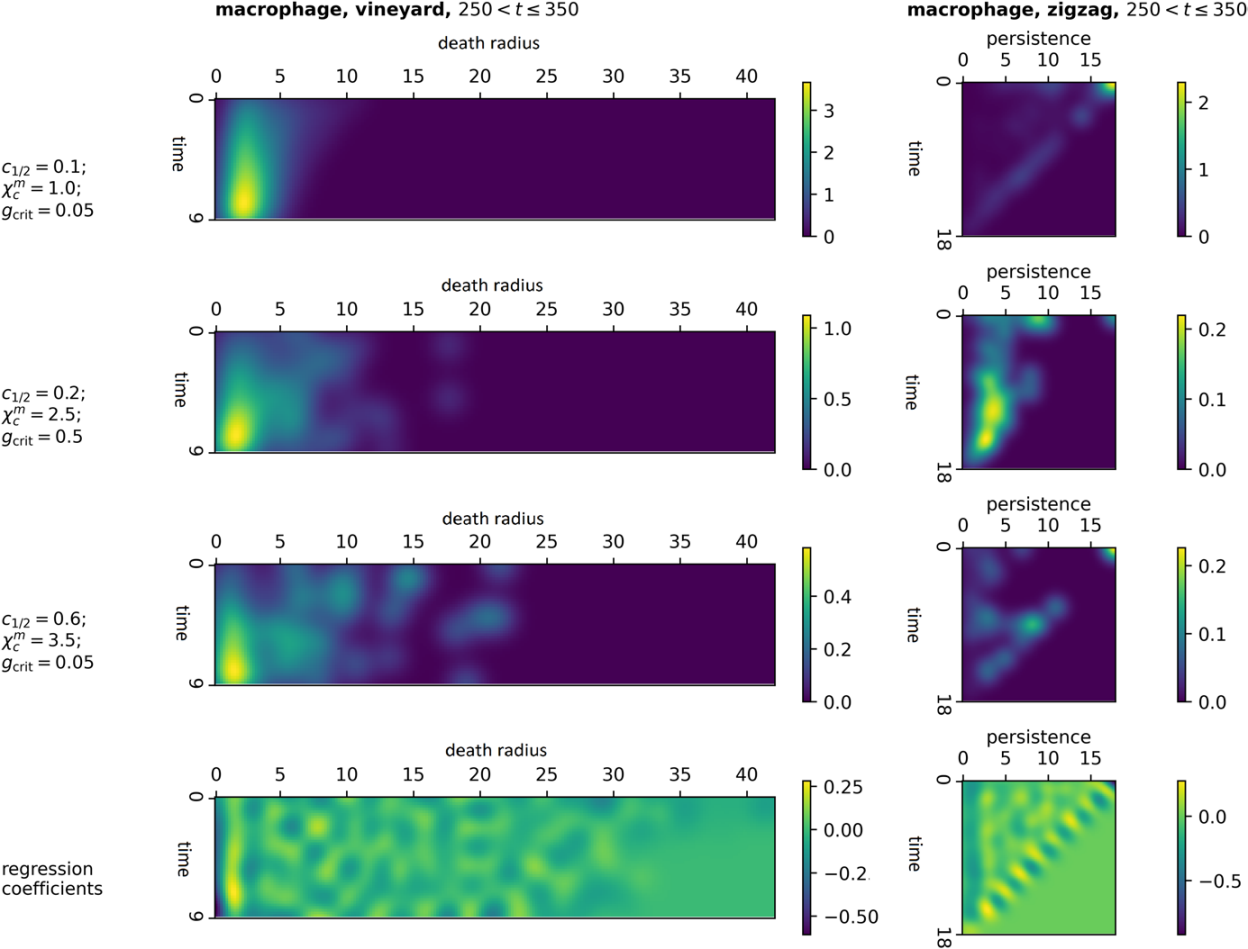
Persistent images of time-dependent filtrations. The first three rows depict features extracted from the equilibrium, elimination, and escape simulations shown in Fig. 3. For each, $\textrm{PI}^{\textrm{vin}}_0(M)$ and $\textrm{PI}^{\textrm{zz}}_0(M)$ are computed using 10 time-steps $250-340$. 1st row: Equilibrium case. 2nd row: Elimination case. 3rd row: Escape case. Last row: Regression coefficients shows the regions of the image which are most important for distinguishing whether or not the simulation would go on to produce a perivascular niche at time $t=500$

The logistic regression coefficients and sample persistence images from the zigzag and vineyard filtrations of macrophage point clouds over the earliest time window, $250 \le t \le 350$ hours, are shown in Fig.  11.

#### Vineyards provide slight advantage over static macrophage clusters $\varvec{\textrm{PI}^{\textrm{VR}}_0(M)}$

Since we envision real-world data to have a limited observational time window, we used a fairly short sequence. At a given time *t*, we compute the Vietoris-Rips persistence diagrams from 10 snapshots of the cell locations at times $t-100, t-90, \dots , t-10$. We stack the Vietoris-Rips persistence diagrams and examine the variation of $\operatorname {PH}_0(\operatorname {VR}(X_t))$ as *t* evolves.

Each trajectory in the image approximates a “vine", tracking the time evolution of a cluster of macrophages, from when the first macrophage emerges from a blood vessel, through changes in composition, scale, spacing, and phenotype, until it merges into a larger cluster or re-enters the blood vessel.

Inspection of the persistence images $\textrm{PI}^{\textrm{vin}}_0(M)$ reveals clear differences in the magnitude and patterns of persistence among the three qualitative behaviours (see Fig. 11). In all three cases, as with $\textrm{PI}^{\textrm{VR}}_0(M)$, most of the relevant features occur at radius $0-2$.

In the equilibrium example (top row), macrophages spaced 1-2 cell diameters apart appear to be the modal type of configuration, while the middle (elimination) case has overall fewer low-persistence features, with a higher proportion of large-scale features. These larger-scale features represent more isolated macrophage clusters. This pattern is even more pronounced in the bottom (pre-escape) case, with fewer clusters overall, and a higher percentage isolated from other clusters.

The noisy pattern in the regression coefficients is difficult to interpret, as the diagrams are more complex, and the spotting in the coefficients may indicate over-fitting. However, the pattern generalises the logistic regression coefficients $\textrm{PI}^{\textrm{VR}}_0(M)$, with a clear weighting on packed features ($0-1$ diameters) to identify non-niche cases, small-scale features ($1-2$ diam.) identifying the niche class, and larger features ($>2$ diam.) trending toward the non-niche class, possibly because of the niche class having fewer features overall. Interestingly, the niche coefficients are highest over timestep 8 ($t=340$), rather than 9 $(t=350)$.

As the accuracy is comparable to $\textrm{PI}^{\textrm{VR}}_0(M)$ until later time steps, we may not have captured some critical dynamics. For example, it appears in our sample images that the length and average isolation of vines may differ between the classes, but this cannot be captured in this vectorisation. Some alternatives are discussed in Sect. 5.2.

#### Zigzags highlight enduring spatial features

Here each $K^t_\varepsilon$ is the static Vietoris-Rips complex at a fixed radius $\varepsilon$, and we use a variable range of ten time steps $(t-100,t-90,t-80,\dots ,t-10)$ from an interval of length 90, ending at $t \in \{250, 300, 350, 400, 450, 500\}$. Along with an intersection complex between each consecutive time step, this gives a total sequence of length 19. At the 10-hour intervals, we typically observe an optimal amount of change: noticeable evolution, but close enough in time to see continuity.

To construct the intersection complex, we utilise the fixed numbering of cells in the simulation to accurately match cells between time steps. This is impossible in real data; for future work, we suggest an estimation methodology via optimal transport matching (see Sect. 5.1). We compute the zigzag barcode for dimension 0 using BATS (Carlsson et al. 2019). A bar with birth time *b* and death time *d* indicates a topological feature that persists through the zigzag filtration between the *b*-th and *d*-th timesteps.

**Linking radius.** The uniform radius $\varepsilon =2$ is selected from a coarse sweep, in the same manner as for the radial filtration in Fig. 9. However, the optimal $\varepsilon$ is significantly larger for the macrophages than the tumour cells, suggesting that macrophages with up to three cells between them should be considered as a functional “cluster".

As macrophages, unlike tumours, move independently, which suggests that whether or not macrophages in close proximity remain in proximity, working in concert, may be relevant to future outcomes.

**Macrophage zigzags –**$\mathbf {\textrm{PI}^{\textrm{zz}}_0(M)}$. We can see clear differences between the $\textrm{PI}^{\textrm{zz}}_0(M)$ in the three example cases (Fig. 11), with the equilibrium simulation (top) exhibiting by far the highest number of macrophage clusters, with over twice as many new macrophage clusters forming during the window that persist through the end. In contrast, the middle image, representing the elimination case before elimination has occurred, shows less overall activity, with the frequent appearance of new features and most features persisting for only a few time steps. The escape case in the bottom $\textrm{PI}^{\textrm{zz}}_0(M)$ shows a much higher proportion of features along the diagonal than the elimination case, with about the same overall activity level. This suggests that the number of macrophage clusters is comparable, but clusters are more static over time.

We examine the logistic regression coefficients to explore the effect of static macrophage clusters. The most distinctive feature of the zigzag regression coefficient array is the diagonal, which represents features that persist through to the end of the zigzag. The diagonal also has a distinct alternating pattern, with negative coefficients at even birth times and positive coefficients at odd ones. As alternating complexes are intersections of the two timesteps on either side, very different behaviour occurs at even (original data-based) and odd (intersection) filtration steps, as shown in Fig. 7. Connected components born from the appearance of new macrophages will appear in the even (data) steps, and new connected components arising from the division of existing connected components will appear in the odd (intersection) step.

The results clearly show a pattern that is not readily apparent from inspecting the images: the niche case is correlated with the division or rearrangement of macrophage clusters from one timestep to the next that remain stable over time, while the non-niche case is correlated with the appearance of new macrophages which remain in the simulation, isolated from other macrophages, for the duration of the time window (see Fig. 11). Additionally, features that have persistence 1, that is, clusters that appear for a single time step, are associated with the non-niche class. Based on our example, this appears to be characteristic of the elimination case.

### Prediction accuracy

**Fig. 12:**
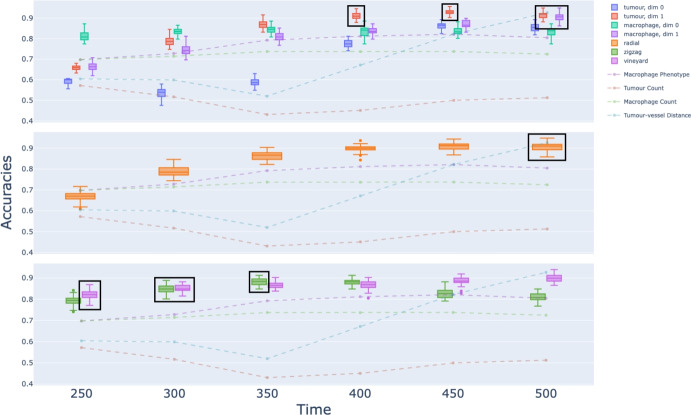
**Accuracy against time.** Accuracy scores in predicting the formation of at least one perivascular niche at 500 hours. Scores are given for the different filtrations based on cell locations at time steps $t=250,300,350,400,450,$ and 500 for the static filtrations, and for each value of *t*, the range $t-100,t-90,\dots , t-20,t-10$ for the dynamic. We compare the four topological methods to various benchmarks associated with simple statistics (dotted lines). The highest scoring methods at each time step are boxed. Top: Vietoris-Rips filtrations. Centre: The tumour radial filtration. Bottom: Zigzag and vineyard filtrations

The percentage of test samples that are correctly predicted to have a perivascular niche or not at time $t = 500$ is given by the accuracy score. We compute the prediction accuracy scores for each method (Fig. 12).

**Comparison to benchmarks.**

All of the topological methods (see Fig. 12) consistently outperform tumour cell counts, which do not accurately predict perivascular niches at any time, and most outperform tumour-vessel distance, which has fairly low accuracy until late time steps.

As benchmarks, we note that the macrophage count and phenotype ratio, which do not involve tumour-vessel information, achieve accuracies of at least 70%, with the phenotype ratio outperforming the macrophage count. All of the topological techniques achieve similar or better accuracies, at much earlier timesteps, except for $\textrm{PI}^{\textrm{VR}}_0(T)$. We hypothesize that like the tumour cell count, $\textrm{PI}^{\textrm{VR}}_0(T)$ has low accuracy due to its difficulty distinguishing the equilibrium and escape cases.

**Highest accuracy uses tumour shape. ** Aside from tumour-vessel distance at time 500 (measurement of which is similar to testing class membership directly), tumour-based PH, including $\textrm{PI}^{\textrm{VR}}_1(T)$ and $\textrm{PI}^{\textrm{rad}}_0(T)$, provide the highest accuracy results in our experiment. $\textrm{PI}^{\textrm{VR}}_1(T)$ and $\textrm{PI}^{\textrm{rad}}_0(T)$ achieve above $90\%$ accuracy for time steps $400 \le t \le 500$, in most cases before perivascular niches appear.

$\textrm{PI}^{\textrm{VR}}_1(T)$ and $\textrm{PI}^{\textrm{rad}}_0(T)$ track the non-compactness of the tumour, including (resp.) holes in the interior and outgrowths from the tumour. The high accuracy scores confirm classic intuition that tumour components and their placement, as well as the regularity of the tumour boundary, including extended outgrowths from the main tumour body, are practical predictors that a perivascular niche is about to form. See Sects. 4.1 and 4.2 for details.

Between the two measures, $\textrm{PI}^{\textrm{VR}}_1(T)$ performs slightly better than $\textrm{PI}^{\textrm{rad}}_0(T)$, although they encode different shape information about the tumour. Computationally, $\textrm{PI}^{\textrm{rad}}_0(T)$ requires only $\operatorname {PH}_0$, which can be more efficient for large data sets than $\textrm{PI}^{\textrm{VR}}_1.$

We emphasise that none of the topological summaries utilise the location of blood vessels or tumour-vessel distance; rather, the measurements are intrinsic to the spatial patterns of macrophages and tumour cells. The closest proxy is the radial filtration $\textrm{PI}^{\textrm{rad}}_0(T)$, which detects distances between tumour cells and the original tumour centre at the midpoint of the simulation. As the blood vessels are located at large radius, there should be high (inverse) correlation between radial birth values and tumour-vessel distance. Nevertheless, $\textrm{PI}^{\textrm{rad}}_0(T)$ outperforms tumour-vessel distance until the final timestep.

**Dynamic methods enable earlier detection.** Among all techniques analysed, macrophage-based homology, including multi-scale clusters $\textrm{PI}^{\textrm{VR}}_0(M)$, zigzag clusters $\textrm{PI}^{\textrm{zz}}_0(M)$, and vineyards of Rips diagrams $\textrm{PI}^{\textrm{vin}}_0(M)$ achieve the highest accuracy in the first half of the time frame studied, at values $250 \le t \le 350.$ By including dynamic topological information in $\textrm{PI}^{\textrm{vin}}_0(M)$ and $\textrm{PI}^{\textrm{zz}}_0(M)$, we are able to achieve a comparable level of accuracy up to 50 time steps earlier than the best-performing static methods. All three macrophage cluster-based methods achieve $80\%$ accuracy 100 time steps earlier than phenotype ratio and 200 time steps earlier than tumour-vessel distance - almost half the lifetime of the simulation.

The strength of these results suggest that given fixed tumour parameters, perivascular niches can be predicted using the arrangements of macrophages alone - above and beyond the number of macrophages, their clustering patterns reflect differences in critical threshold and chemotactic sensitivity.

## Conclusion and future work

In order to better understand the qualitative behaviour of tumour-immune interactions, we studied a series of topological summaries which represent the shape of a point cloud as a persistence image. We tested these methods on an agent-based model, to try to predict which simulations would go on to form a perivascular niche, and which would not.

We compared the corresponding topological vectorisations to alternative, benchmarking features (e.g., cell counts, macrophage phenotype, and distance from tumour to blood vessel). We found persistence images, encoding the geometry and topology of the point clouds of tumour and macrophage cells, performed exceptionally well at predicting the future development of perivascular niches.

At early stages, starting at the halfway point of the simulation $t=250$, topological summaries based on the macrophage point cloud were able to predict perivascular niches with $80\%$ accuracy, matching the accuracy of macrophage phenotype ratio over 100 time steps earlier. Dynamic methods $\textrm{PI}^{\textrm{vin}}_0(M)$ and $\textrm{PI}^{\textrm{zz}}_0(M)$ allowed for slightly earlier classification than static macrophage multi-scale clusters, by capturing the topological evolution of the point cloud of macrophages over the preceding 90 h.

At time step 400 h, topological summaries based on holes and tortuosity in the tumour point cloud achieves $90\%$ accuracy in the prediction of perivascular niche formation.

Based on their performance, we can confirm intuition that 1-dimensional features, or holes, inside the tumour point cloud and irregularity of the tumour boundary are highly predictive. The logistic regression coefficients contained even richer detail on the geometric features relevant for each class, showing that it is larger holes, and tumour outgrowths of a particular length, that are most indicative of potential for a perivascular niche, with macrophage accumulation around the boundary of a tumour indicating a lower likelihood of niche formation.

These results lead naturally to future research directions, including the application of the topological techniques to real data, as described in Sect. 5.1, the extension of the methods to more sophisticated topological summaries, as described in Sect. 5.2, and the investigation of biological hypotheses arising from the topological class differences.

### Adapting the methods to biological data

The true strengths and limitations of any method only become apparent when they are evaluated in real-world applications. We believe, nonetheless, that the pipelines we have developed can be easily applied to other synthetic data as well as adapted to other spatio-temporal experimental/clinical data. While most of our topological constructions could be applied to real data sets without modification, the zigzag and radial complexes require some adaptation. The radial complex, for example, requires a choice of basepoint. In our analysis, we fixed the basepoint at the centre of the simulation, but in imaging data from cancer, the tumour’s centre of mass could be used similarly, as in application of radial filtration to tumour vascular networks in Stolz et al. (2022). Further, both the radial and zigzag require a fixed linkage radius, which depends on the size and density of the cells.

To construct the intersection complex for the zigzag, we used information from the simulations (the identities of each autonomous cell) that would only be available in live-tracking data, but not in other snapshot capture experiments. A point is present in the intersection complex if the corresponding cell is present in both timesteps, even if it has moved significantly. Similarly, higher simplices are present if they are present in both $K^t_\varepsilon$ and $K^{(t+10)}_\varepsilon$, that is, if the cells remain in (or return to) proximity within the interval. Since real data will typically not contain individual cell trajectories, it may not be possible to determine which simplices are new, and which have moved. We propose that cell identity would be best approximated by continuity, finding *M* which minimises $\inf _M \left( \sum _{(m_1,m_2)\in M}||m_1-m_2||^2\right) ,$ where $M = \{(m_1,m_2)\}$ is a matching of $M_1 = M_t \cup V$ and $M_2 = M_{t+10}\cup V$ which gives a bijection between $M_1$ and $M_2$, but with arbitrary multiplicity on blood vessel set *V*. Implementation of this method on our synthetic data yielded similar results in preliminary testing, but we postpone further development of this approach to future research.

Finally, data sets of different sizes and/or scales would require normalization of the persistence images. We have chosen to vectorise our diagrams into persistence images in part for the ease of normalization, as images can be cropped, padded, or scaled easily. As the local behaviour of cells may be comparable for areas (and tumours) of any size, we recommend normalising the images to the same birth axis.

### Further methods and analysis

As several of our methods individually outperformed the benchmarks, with different features and strengths, we naturally would consider combining several vectorisations in order to increase predictive accuracy. Our results suggest that a macrophage-based clustering, such as $\textrm{PI}^{\textrm{vin}}_0(M)$ (or $\textrm{PI}^{\textrm{VR}}_0(M)$ where fine time steps are not available), combined with tumour $\textrm{PI}^{\textrm{VR}}_1(T)$ or $\textrm{PI}^{\textrm{rad}}_0(T)$, would be particularly powerful when the stage of development is not known.

It is also natural to consider combining filtrations into a multiparameter persistence module. Multipersistence has already been applied to study spatial patterns of immune cells in tumours in Vipond et al. (2021). The radial filtration, for example, could be generalised to avoid choosing a linkage radius, so that the complex is simultaneously filtered on distance from the centre and scale, as in the Vietoris-Rips complex. We may also bypass the same issue of having to choose an $\varepsilon$ in the zigzag filtration by studying spatio-temporal data with an interlevel-Rips trifiltration, as in Kim and Mémoli (2021). To our knowledge, no software exists for such analyses.

A second obvious direction would be the extension to 3-dimensional models and data. Although we developed these techniques for a two-dimensional spatial model, the methods described can also be applied to 3-dimensional point clouds with little modification to the computational pipeline. Indeed, many of the works cited analyse 3-d data with similar techniques, and we expect our results to extend naturally. In 3-d data it is possible to identify higher homology groups, representing loops and enclosed voids in the 3-d filtrations. These higher order features may provide additional classifying power.

Finally, we note that persistence diagrams can be vectorised in several different ways (Ali et al. 2023), and persistence images are one choice among many others such as *persistence landscapes* (Bubenik 2012) and *CROCKER plots* (used in Topaz et al. 2015). Persistence flamelets have also been suggested as a robust summary for time-varying data; they could serve as a reasonable substitute for our vineyard vectorisation (Padellini and Brutti 2023). While we anticipate that persistence images are likely to offer the most robust results for this type of classifier, this remains to be tested.

## Data Availability

Our code and data can be found at https://github.com/Pyxidatol-C/ABM-TDA.
